# Sex and Biomarkers in FTD

**DOI:** 10.1002/alz70857_100827

**Published:** 2025-12-24

**Authors:** Sterre C.M. de Boer, Chiara Fenoglio, Andrea Arighi, Lina Riedl, Ishana Rue, Ramon Landin‐Romero, Sophie Matis, Zac Chatterton, Glenda M Halliday, Janine Diehl‐Schmid, Olivier Piguet, Inge M.W. Verberk, Charlotte E. Teunissen, Simon Ducharme, Sven J van der Lee, Yolande A.L. Pijnenburg, Daniela Galimberti

**Affiliations:** ^1^ Alzheimer Center Amsterdam, Department of Neurology, Amsterdam Neuroscience, Vrije Universiteit Amsterdam, Amsterdam UMC, Amsterdam, Netherlands; ^2^ University of Milan, Fondazione Cà Granda, IRCCS Ospedale Policlinico, Milan, Italy; ^3^ Department of Biomedical, Surgical and Dental Sciences. University of Milan, Milan, Milan, Italy; ^4^ University of Milan, Fondazione Cà Granda, IRCCS Ospedale Policlinico, Milan, Milan, Italy; ^5^ Technical University of Munich, School of Medicine, Munich, Germany; ^6^ Douglas Mental Health University Institute, McGill University, Montreal, QC, Canada; ^7^ The University of Sydney, Brain and Mind Centre, Sydney, NSW, Australia; ^8^ The University of Sydney, Brain & Mind Centre, Camperdown, NSW, Australia; ^9^ The University of Sydney, Sydney, NSW, Australia; ^10^ Inn‐Salzach‐Klinikum, Wasserburg, Germany; ^11^ The University of Sydney, School of Psychology and Brain & Mind Centre, Sydney, NSW, Australia; ^12^ Amsterdam Neuroscience, Neurodegeneration, Amsterdam, Netherlands; ^13^ Neurochemistry Laboratory, Department of Clinical Chemistry, Amsterdam UMC, location VUmc, Amsterdam, Netherlands; ^14^ Alzheimer Center Amsterdam, Department of Neurology, Vrije Universiteit Amsterdam, Amsterdam UMC location VUmc, Amsterdam, The Netherlands, Amsterdam, Netherlands; ^15^ Alzheimer Center Amsterdam, Neurology, Vrije Universiteit Amsterdam, Amsterdam UMC location VUmc, Amsterdam, Netherlands; ^16^ Montreal Neurological Institute, McGill University, Montreal, QC, Canada; ^17^ McConnell Brain Imaging Centre, Montreal Neurological Institute, McGill University, Montreal, QC, Canada; ^18^ Amsterdam Neuroscience, Amsterdam, Netherlands; ^19^ Section Genomics of Neurodegenerative Diseases and Aging, Department of Human Genetics, Vrije Universiteit Amsterdam, Amsterdam, Noord‐Holland, Netherlands; ^20^ Amsterdam Neuroscience, Amsterdam UMC, Amsterdam, Netherlands; ^21^ Alzheimer Center Amsterdam, Neurology, Vrije Universiteit Amsterdam, Amsterdam UMC location VUmc, Amsterdam, Amsterdam, Netherlands; ^22^ Fondazione IRCCS Ca’ Granda, Ospedale Policlinico, Neurodegenerative Diseases Unit, Milan, Italy; ^23^ Department of Biomedical, Surgical and Dental Sciences, University of Milan, Milan, Italy

## Abstract

**Background:**

Sex‐linked differences in sporadic behavioral variant frontotemporal dementia (s‐bvFTD) are increasingly recognized. Research suggests that females with bvFTD exhibit greater atrophy in affected brain regions than males, despite similar clinical severity. Given that brain atrophy correlates with elevated markers of neurodegeneration, such as neurofilament light (NfL) and glial fibrillary acidic protein (GFAP), we hypothesized that these biomarkers differ between sexes.

**Method:**

Serum NfL and GFAP levels were measured using Simoa in 357 participants (275 with s‐bvFTD, 82 with primary psychiatric disorders (PPD)). Biomarker levels were compared between sexes, followed by linear regression analyses stratified by diagnostic group. In a subset of s‐bvFTD participants (47 females, 105 males) with available Z‐scored cognitive composite scores (including global cognition, executive function, attention and working memory), we performed linear regression using a residuals approach (with age at blood sample (AAS), education, and Z‐scored NfL or GFAP as predictor) to assess cognitive reserve.

**Result:**

Females with s‐bvFTD had significantly higher NfL and GFAP levels than males (both *p* <0.05), whereas no sex differences were observed in the PPD group. Within s‐bvFTD, higher AAS (β=0.015, 95% CI [0.006‐0.023]) and female sex (β=0.223, 95% CI [0.071‐0.375]) were associated with higher logNfL. In PPD, higher AAS (β=0.030, 95% CI [0.019‐0.042]) correlated with higher logNfL, but no sex effect was observed (β=‐0.092, 95% CI [‐0.265‐0.082]). For GFAP, higher AAS (β=0.030, 95% CI [0.023‐0.037]) and female sex (β=0.305, 95% CI [0.183‐0.427]) were associated with higher logGFAP in s‐bvFTD. In PPD, only AAS (β=0.026, 95% CI [0.012‐0.040]) was significant, without a sex effect (β=0.033, 95% CI [‐0.177‐0.243]). Composite scores were lower in females (female=‐0.24 vs. male=0.003), though not significantly (*p* >0.05). Residuals of linear regression did not differ by sex for both GFAP and NfL (*p* >0.05). However, NfL (but not GFAP) predicted a steeper cognitive decline in males (β=‐0.15, *p* <0.05) than in females (β=‐0.12, *p* >0.05).

**Conclusion:**

Females with s‐bvFTD exhibited significantly higher NfL and GFAP levels. This sex difference is not explained by differences in cognitive reserve. Given that NfL plateaus as disease progresses, these findings suggest that females with s‐bvFTD may present at a more advanced disease stage upon clinical presentation.

